# LINC00673 is activated by YY1 and promotes the proliferation of breast cancer cells via the miR-515-5p/MARK4/Hippo signaling pathway

**DOI:** 10.1186/s13046-019-1421-7

**Published:** 2019-10-17

**Authors:** Kun Qiao, Shipeng Ning, Lin Wan, Hao Wu, Qin Wang, Xingda Zhang, Shouping Xu, Da Pang

**Affiliations:** 1 Department of Breast Surgery, Harbin Medical University Cancer Hospital, 150 Haping Road, Harbin, 150086 China; 2 Heilongjiang Academy of Medical Sciences, 157 Baojian Road, Harbin, 150086 China

**Keywords:** YY1, LINC00673, miR-515-5p, MARK4, Hippo signaling pathway, Cell proliferation, Breast cancer

## Abstract

**Background:**

An increasing number of studies have shown that long noncoding RNAs (lncRNAs) play essential roles in tumor initiation and progression. LncRNAs act as tumor promoters or suppressors by targeting specific genes via epigenetic modifications and competing endogenous RNA (ceRNA) mechanisms. In this study, we explored the function and detailed mechanisms of long intergenic nonprotein coding RNA 673 (LINC00673) in breast cancer progression.

**Methods:**

Quantitative real-time PCR (qRT-PCR) was used to examine the expression of LINC00673 in breast cancer tissues and in adjacent normal tissues. Gain-of-function and loss-of function experiments were conducted to investigate the biological functions of LINC00673 in vitro and in vivo. We also explored the potential role of LINC00673 as a therapeutic target using antisense oligonucleotide (ASO) in vivo*.* RNA sequencing (RNA-seq), dual-luciferase reporter assays, chromatin immunoprecipitation (ChIP) assay, and rescue experiments were performed to uncover the detailed mechanism of LINC00673 in promoting breast cancer progression.

**Results:**

In the present study, LINC00673 displayed a trend of remarkably increased expression in breast cancer tissues and was associated with poor prognosis in breast cancer patients. Importantly, LINC00673 depletion inhibited breast cancer cell proliferation by inhibiting the cell cycle and increasing apoptosis. Furthermore, ASO therapy targeting LINC00673 substantially suppressed breast cancer cell proliferation in vivo. Mechanistically, LINC00673 was found to act as a ceRNA by sponging miR-515-5p to regulate MARK4 expression, thus inhibiting the Hippo signaling pathway. Finally, ChIP assay showed that the transcription factor Yin Yang 1 (YY1) could bind to the LINC00673 promoter and increase its transcription *in cis.*

**Conclusions:**

YY1-activated LINC00673 may exert an oncogenic function by acting as a sponge for miR-515-5p to upregulate the MARK4 and then inhibit Hippo signaling pathway, and may serve as a potential therapeutic target.

## Background

Breast cancer is the most commonly diagnosed cancer and the leading cause of cancer death among women [1]. Despite marked improvements in diagnosis and treatment, breast cancer remains a dangerous disease among women globally [2]. Early detection is still of the utmost importance in reducing breast cancer mortality but is hampered by a lack of effective diagnostic biomarkers. Thus, it is necessary to elucidate the molecular mechanisms of breast cancer to improve its diagnosis and to provide tailored molecularly stratified therapy.

Long noncoding RNAs (lncRNAs) are defined as transcripts longer than 200 nucleotides that lack an extended open reading frame and thus do not code for proteins [3–5]. LncRNA expression is usually tissue-restricted and developmentally regulated and can change under specific pathological conditions. Indeed, several lncRNAs have been shown to influence behaviors in human cancers, such as uncontrolled proliferation, as well as metastasis formation, and it has been suggested that lncRNAs can act as oncogenes or tumor suppressors by interfering with different signaling pathways [6, 7]. Mechanistically, lncRNAs may influence the function of transcriptional complexes, modulate chromatin structures by serving as scaffolds between proteins, or act as microRNA sponges [4, 8]. Long intergenic nonprotein coding RNA 673 (LINC00673) was first annotated as a long intergenic noncoding RNA (lincRNA) on chromosome 17q24.3 [9]. Recent studies have reported that LINC00673 is a potential tumor suppressor whose germline variation is associated with pancreatic cancer risk [10]. Conversely, LINC00673 was identified to play a role in tumorigenesis in a variety of malignancies [11–15]. A rapidly growing number of studies have suggested that cytoplasmic lncRNAs are essential mediators of intracellular signaling pathways and are involved in regulating mRNA stabilization and transport as well as microRNA sponging [16–18]. Although LINC00673 has been implicated in the regulation of breast cancer cell metastasis via the modulation of epithelial-mesenchymal transition (EMT) in breast cancer [19], it remains to be seen whether LINC00673 acts as a competing endogenous RNA (ceRNA) to promote cell proliferation in breast cancer.

The Hippo signaling pathway, a critical pathway in tumorigenesis, is evolutionarily conserved and has multiple biological functions in development, homeostasis, and the regeneration of tissues and organs [20]. The dysregulation of the Hippo signaling pathway occurs in many human tumors, including glioma, colorectal cancer, and breast cancer [21]. The major effectors of this pathway are the related transcriptional coactivators, Yes-associated protein 1 (YAP) and Transcriptional coactivator with PDZ-binding motif (TAZ), which, in association with various transcription factors such as transcriptional enhanced associate domains (TEADs), induce a growth-promoting gene expression program to regulate cell proliferation, apoptosis, and differentiation [22–24].

Antisense oligonucleotides (ASOs) are short, single-stranded, synthetic analogues of natural nucleic acids designed to specifically bind to a target messenger RNA (mRNA) in a sequence-specific manner through the Watson–Crick base-pair interactions [25]. They were first discovered to influence RNA processing and modulate protein expression over two decades ago [26–28]. Furthermore, ASOs can be designed to target genes associated with disease pathogenesis, including cancer,especially those that are not amenable to small-molecule or antibody inhibition [29–31]. With the rapid development of improved next-generation ASOs toward clinical application, a large amount of work is currently being carried out to develop chemical modifications and vehicles that will improve ASOs delivery and target engagement.

In the current study, we showed that LINC00673 was upregulated in breast cancer tissues compared with adjacent normal tissues and that elevated LINC00673 levels were associated with poor prognosis in breast cancer patients. Moreover, the knockdown of LINC00673 significantly inhibited breast cancer cell proliferation in vitro and in vivo. Furthermore, our study verified that antisense oligonucleotide therapy targeting LINC00673 substantially suppressed breast cancer progression in vivo. Mechanistically, LINC00673 promotes tumor proliferation by sponging miR-515-5p to regulate MARK4 and then inhibits the Hippo signaling pathway. Finally, we determined that the transcription factor Yin Yang 1 (YY1) could activate LINC00673 transcription in breast cancer cells. In conclusion, our study provides new information for breast cancer therapy, and LINC00673 may be a potential therapeutic target in breast cancer.

## Methods

### Patients and specimens

Breast cancer specimens and adjacent noncancerous tissues were obtained from Harbin Medical University Cancer Hospital, and patients with a histological diagnosis of breast cancer who had received neither chemotherapy nor radiotherapy before surgical resection were recruited for the present study between 2010 and 2014. This study conformed to clinical research guidelines and was approved by the research ethics committee of Harbin Medical University Cancer Hospital. We obtained written informed consent from all patients.

### Cell culture and treatments

Normal mammary cells (MCF-10A), breast cancer cell lines (MDA-MB-231, MDA-MB-453, MDA-MB-468, Hs-578 T, MCF-7, T-47D and BT-549) and the 293 T cell line were obtained from the Chinese Academy of Sciences Cell Bank and Cellbio (China) and were cultured according to the suppliers’ instructions.

### RNA isolation and quantitative real-time PCR (qRT-PCR)

Total RNA was extracted using Trizol Reagent (Invitrogen, CA, USA) according to the manufacturer’s instructions. First-strand complementary DNA (cDNA) was prepared with a Transcriptor First Strand cDNA Synthesis Kit (Cat# 04897030001, Roche, USA). Real-time PCR was performed using FastStart Universal SYBR Green Master (ROX) (Cat#04913914001, Roche) on a 7500 Fast Real-Time PCR system (ABI, USA). For the quantification of gene expression, we used the 2^-ΔΔCt^ method. GAPDH or U6 expression was used for normalization. The primer sequences were synthesized by Genepharma (Shanghai, China). All the primer sequences are available in Additional file 3: Table S1.

### Subcellular fractionation

Nuclear/cytoplasmic fractionation was performed by using NE-PER™ Nuclear and Cytoplasmic Extraction Reagents (Cat#78835, Thermo Fisher) according to the manufacturer’s protocols. U1 was used as a nuclear control, while GAPDH was used a cytoplasmic control.

### Antisense oligonucleotide (ASO) and cell transfection

ASO was designed and synthesized by Integrated DNA Technologies (Cat#257249069, USA). To determine the in vitro ASO interference efficiency, cells were seeded in six-well plates and transfected with 1.4 μg of the ASO premixed with 5.6 μl of DOTAP Liposomal Transfection Reagent (Cat#11202375001, Roche, USA). For the in vitro ASO proliferation assay, cells were seeded in 96-well plates and transfected with 5 μM of the ASO premixed with 1.0 μl DOTAP Liposomal Transfection Reagent. A total concentration of 0 μM of the ASO was used as the control. Lentiviruses expressing YY1, LINC00673 and sh-LINC00673 and controls were constructed by Genechem (Shanghai, China). Concentrated viruses were used to infect 5 × 10^5^ cells in a 6-well plate with 4–6 μg/ml Polybrene. The infected cells were then subjected to selection with 1 μg/ml puromycin (Cat#540411, Calbiochem, USA) for 1 weeks. Stable overexpression cell lines or knockdown cell lines were identified using qRT-PCR or western blotting. Small interfering RNA (siRNA) duplex oligonucleotides targeting human MARK4 and LINC00673 and controls were synthesized by Genepharma (Shanghai, China). The miR-515-5p mimic and inhibitor were purchased from Ribobio (Guangzhou, China). Cell transfections were performed using Lipofectamine 2000 (Invitrogen, Carlsbad, CA, USA) according to the manufacturer’s instructions. At 48 h post transfection, cells were harvested for qRT-PCR or western blot analyses. The ASO, shRNA and siRNA sequences are listed in Additional file 3: Table S1.

### Cell proliferation assays

The CCK-8 assay and the colony formation assay were performed to test cell proliferation. Briefly, for the CCK-8 assay, 1 × 10^3^ cells were cultured in a 96-well plate at 37 °C. After 10 μl CCK-8 solution was added to each well, plates were incubated at 37 °C for 1 h. The cell proliferation curves were plotted by measuring the 450 nm absorbance at each indicated time point. Experiments were performed in triplicate. For the colony formation assay, cells were exposed to the indicated treatments, were seeded in 6-well plates and were cultured for 2 weeks. Cell colonies were washed with phosphate-buffered saline (PBS), fixed with 4% paraformaldehyde, stained with 0.1% crystal violet and imaged using an optical microscope.

### TUNEL assay and flow cytometry

To detect apoptosis in sections of tumor tissues, a TUNEL assay was performed according to the manufacturer’s instructions (Cat#11684795910, Roche), as previously described [32]. Apoptosis was further examined using a FITC Annexin V Apoptosis Detection Kit I (BD Biosciences). Briefly, cells were harvested and washed twice with ice-cold PBS and were stained with PE Annexin V and 7-AAD (BD Bioscience, San Jose, CA, USA) for 15 min at room temperature. For the analysis of the cell cycle, the transfected cells were stained and fixed in ice-cold 75% ethanol overnight at 4 °C. After fixation, the cells were washed and resuspended twice in PBS and were then incubated with propidium iodide (BD Bioscience) and RNase for 30 min at room temperature. The cells were then analyzed using a FACS Calibur flow cytometer (BD Biosciences, San Jose, CA, USA).

### Hematoxylin-eosin (H&E) staining

Organs were fixed in 4% paraformaldehyde at 4 °C overnight. Then, the tissues were embedded in paraffin and cut into 5-μm slices. Deparaffinized and rehydrated sections were stained with H&E. Then, the stained sections were observed under a microscope (Olympus, Tokyo, Japan).

### Western blotting and antibodies

Cells were lysed with RIPA extraction reagent (Beyotime) supplemented with a protease inhibitor cocktail (Roche). Proteins were separated by 6–15% SDS-PAGE, transferred to 0.22 mm polyvinylidene fluoride membranes (Millipore), and then incubated with antibodies. The bands on the blots were captured by using an Odyssey Infrared Imaging System (LI-COR Biosciences) and were quantified with Odyssey v1.2 software (LI-COR Biosciences). GAPDH and Tubulin were used as the internal controls. Antibodies against the following proteins were used: Bax (Cell Signaling Technology Cat#5023,1:1000), Bcl-2 (Cell Signaling Technology Cat#4223,1:1000), MARK4 (Cell Signaling Technology Cat#4834,1:1000), YY1 (Cell Signaling Technology Cat#46395,1:1000), phospho-YAP/TAZ sampler kit (Cell Signaling Technology Cat#52420), Cyclin D1 (Cell Signaling Technology Cat#2978,1:1000), GAPDH (Cell Signaling Technology Cat#5174,1:1000), and Tubulin (Santa Cruz Biotechnology Cat#sc-73,242,1:1000). Alexa Fluor® 800 goat anti-mouse (LI-COR Biosciences, Cat#926–32,210,1:10000) or anti-rabbit (LI-COR Biosciences, Cat#926–32,211,1:10000) was used as a secondary antibody. Protein bands were quantified using Odyssey v1.2 software (LI-COR Biosciences), and GAPDH and Tubulin were used as the internal controls.

### Animal experiments

The animal study protocol was approved by the Institutional Animal Care and Use Committee of Harbin Medical University Cancer Hospital and was performed in accordance with the Guide for the Care and Use of Beijing Vital River Laboratory Animal Technology Co, Ltd. (Beijing, China). Mouse xenograft models were established using 4-week-old BALB/c nude female mice. MDA-MB-231 cells labeled with luciferase (1 × 10^6^; stably expressing sh-LINC00673, or sh-NC) were harvested and resuspended in 0.9% normal saline and in 0.2 mL 25% phenol red-free Matrigel (Cat#356234, Corning). Then, cells were directly injected into the mammary fat pads of the mice. The tumor volume was calculated using the formula V = length × width × width/2. ASOs were delivered by intravenous tail injection at a dose of 50 mg/kg twice a week when the tumors reached approximately 100 mm^3^. The control mice received 0.9% normal saline. For bioluminescence imaging, tumor-bearing mice were injected with luciferin substrate (150 mg/kg) into the tail vein and were imaged on an In-Vivo Imaging System Fx Pro (BRUKER, Germany) 30 min after injection. The tumor weight was measured at the endpoint of the study. The eyeballs of the mice were extracted, and blood samples were collected for biochemical analysis using an FDC 500iVC automatic biochemical analyzer (FUJIFILM, Japan).

### Luciferase reporter assays

HEK293T cells were seeded at 5 × 10^4^ cells/well in 24-well plates and were cultured overnight. On the next day, the cells were cotransfected with pmirGLO-LINC00673-WT, pmirGLO-LINC00673-MUT, pmirGLO-MARK4–3’UTR-WT, pmirGLO-MARK4–3’UTR-MUT reporter plasmids (Genechem, Shanghai), NC mimics or miR-515-5p mimics. Twenty-four hours post transfection, cells were lysed using passive lysis buffer (Promega), and the luciferase activity was measured by a GloMax20/20 Luminometer (Promega) using the Dual-Luciferase Reporter Assay System (Promega) and was normalized to the renilla luciferase activity.

### Chromatin immunoprecipitation (ChIP)

ChIP assays were performed using a commercially available kit (Beyotime) according to the manufacturer’s protocol. Briefly, cells were cross-linked with 1% formaldehyde and were sonicated on ice to create 200–500 bp fragments. Stained chromatin was cultured overnight with an anti-YY1 antibody (Cat#46395, CST, USA,1:50) or IgG (sc-2027, Santa Cruz Technology) as an isotype control. The precipitated chromatin DNA was recovered and analyzed by qRT-PCR. The primer sequences are shown in Additional file 3: Table S1.

### RNA sequencing (RNA-seq)

RNA preparation, library construction and sequencing were performed on the BGISEQ-500 platform at the Beijing Genomics Institute (BGI, Shenzhen, China). Statistical analysis was performed, and differentially expressed genes (DEGs) were selected that met the criteria of a fold change ≥ 1.3 and *P* ≤ 0.05. RNA-seq data were deposited in the Gene Expression Omnibus (Accession no. GSE133331, https://www.ncbi.nlm.nih.gov/geo/query/acc.cgi?acc=GSE133331).

### Bioinformatics analysis

The Cancer Genome Atlas (TCGA) (https://tcga-data.nci.nih.gov) and the MiTranscriptome (http://www.mitranscriptome.org) [33] breast cancer databases were used to compare the expression of LINC00673 in normal and breast cancer tissues by using R software. The LncBook (https://bigd.big.ac.cn/lncbook) [34] and TargetScan (http://www.targetscan.org/) [35] databases were used to examine putative miRNA interactions between LINC00673 and MARK4. The YY1 binding motif in the promoter region of LINC00673 was identified by JASPAR (http://jaspar.genereg.net/) [36] and TRANSFAC (http://gene-regulation.com/).

### Statistical analysis

Student’s t-tests were used for comparisons between experimental and control conditions, and one-way ANOVA was used for multiple group comparisons. The Chi-square test was used to assess correlations between LINC00673 expression and the clinicopathological features of breast cancer patients. The correlation between LINC00673 and MARK4 expression was analyzed using Spearman’s correlation test. The survival curves were constructed with the Kaplan-Meier method and were compared with the log-rank test. Statistical analysis was performed using GraphPad Prism 7. The results are expressed as the mean ± the standard deviation (SD) of at least three independent experiments. *P* < 0.05 was considered statistically significant.

## Results

### LINC00673 is upregulated in breast cancer tissues and cell lines

**Fig. 1:**
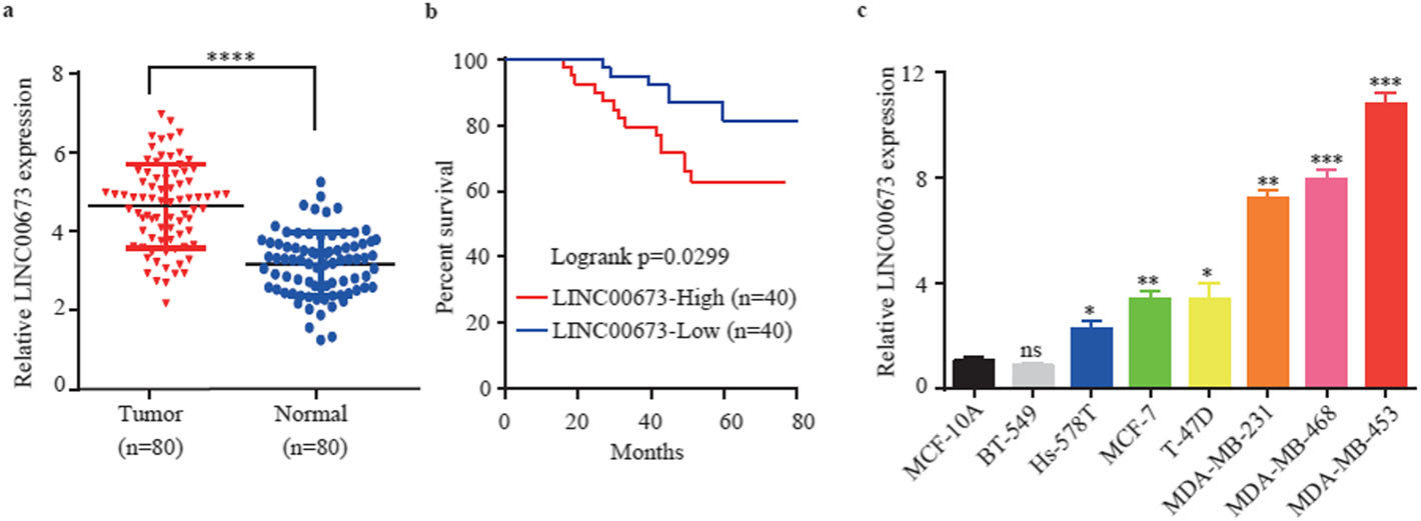
LINC00673 is upregulated in breast cancer tissues and cell lines. **a** LINC00673 expression was determined by qRT-PCR in 80 paired breast cancer tissues and adjacent normal tissues. **b** Kaplan-Meier OS curves are illustrated based on the LINC00673 level. The median expression level was used as the cutoff. **c** Expression of LINC00673 was verified by qRT-PCR in breast cancer cell lines and normal cell lines. The data are presented as the mean ± the SD of three independent experiments. *P < 0.05; ** *P* < 0.01; *** *P* < 0.001; ****P < 0.0001; and ns, not significant

To investigate the expression of LINC00673 in breast cancer, the MiTranscriptome database was applied, and LINC00673 was expressed at higher levels in breast cancer samples than in normal breast tissues (Additional file 1: Figure S1a). This finding was further validated by TCGA breast cancer data showing that LINC00673 was upregulated in breast cancer tissues compared to normal tissues (Additional file 1: Figure S1b). To further validate this result, we investigated LINC00673 expression in 80 pairs of primary breast cancer tissues and their corresponding adjacent tissues. These results showed that LINC00673 expression was markedly increased in tumor tissues compared with normal tissues (Fig. 1a). When stratified by the median expression of LINC00673, patients with high LINC00673 expression had significantly shorter overall survival (OS) than those with low LINC00673 expression (Fig. 1b). In addition, we examined the correlation of LINC00673 expression with patients’ clinicopathological characteristics in breast cancer. LINC00673 expression was positively correlated with tumor size (*P* = 0.024), and Ki67 status (*P* = 0.019). However, no significant association was found between LINC00673 expression and age, lymph node metastasis (LNM), tumor-node-metastasis (TNM) stages, Her-2 status, or ER/PR status (Table 1). Next, we investigated the expression of LINC00673 in breast cancer cell lines, which included MDA-MB-231, MDA-MB-453, MDA-MB-468, Hs-578 T, MCF-7, T-47D, BT-549 and Normal mammary cells MCF-10A. These results showed that the expression of LINC00673 was significantly upregulated in the more aggressive MDA-MB-453, MDA-MB-468 and MDA-MB-231 cell lines compared with MCF-10A cells (Fig. 1c). Therefore, we hypothesize that high expression of LINC00673 may be involved in tumor cell proliferation and may be an oncogene in breast cancer.

**Table 1:** Correlation between LINC00673 expression and the clinicopathological features of breast cancer

Characteristics	No. (*n* = 80)	LINC00673 expression		*P*-value
		Low (*n* = 40)	High (*n* = 40)	
Age				
< 50	38	24	14	0.446
≥ 50	42	23	19	
Tumor size				
≤ 2 cm	31	20	11	0.024^*^
> 2 cm	49	19	30	
LNM				
Negative	27	17	10	0.097
Positive	53	23	30	
TNM stage				
I-II	74	39	35	0.089
III-IV	6	1	5	
ER expression				
Negative	29	16	13	0.485
Positive	51	24	27	
PR expression				
Negative	45	23	25	0.648
Positive	32	17	15	
Her-2 expression				
Negative	44	24	20	0.368
Positive	36	16	20	
Ki67 expression				
≤ 14%	28	19	9	0.019^*^
> 14%	52	21	31	

*LNM* lymph node metastasis, *P < 0.05

### LINC00673 promotes breast cancer cell proliferation in vitro

**Fig. 2:**
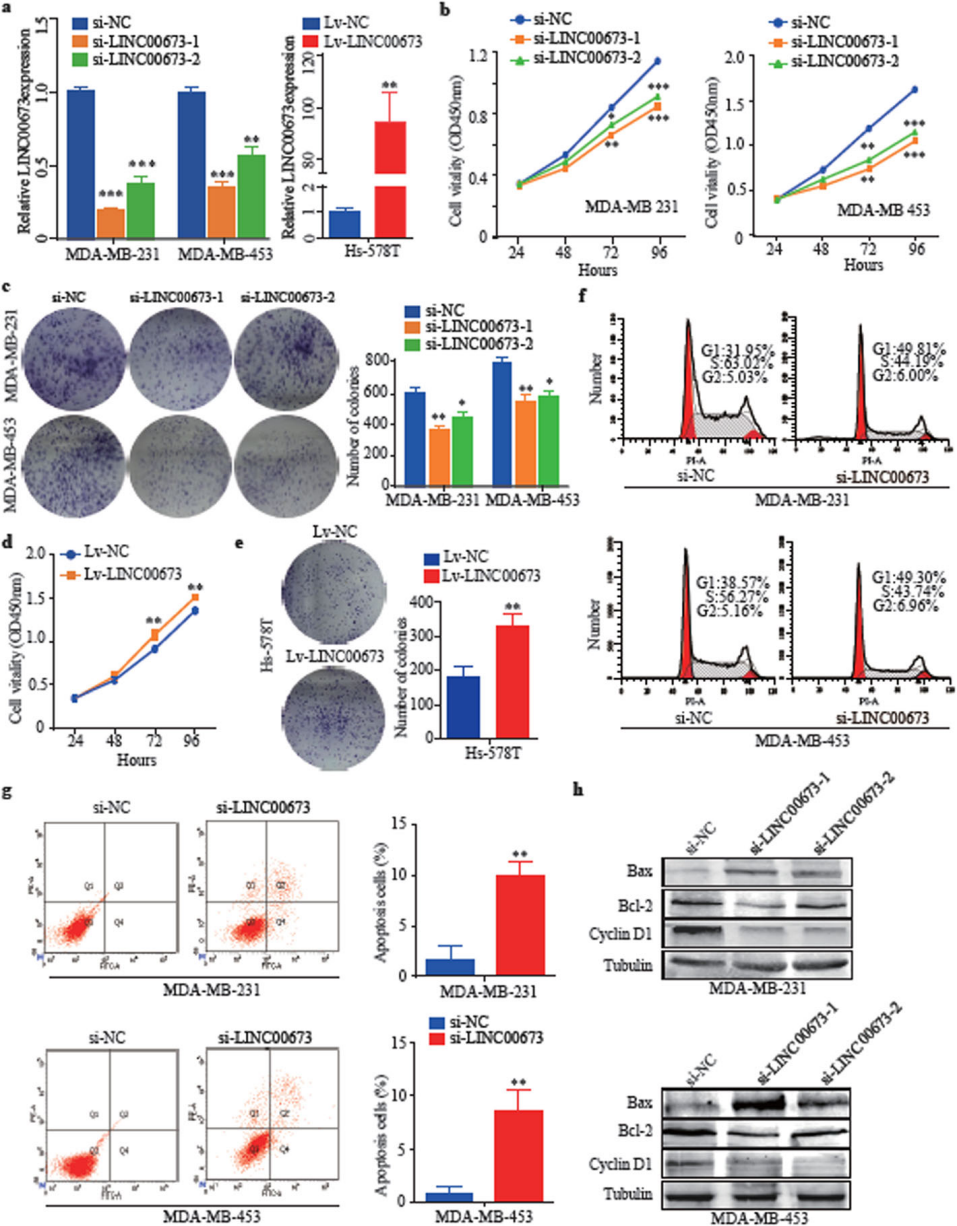
LINC00673 promotes breast cancer cell proliferation in vitro. **a** The LINC00673 expression level in MDA-MB-231, MDA-MB-453, and Hs-578 T breast cancer cells transfected with si-LINC00673 or Lv-LINC00673. **b** CCK-8 assays were performed to determine the cell viability in MDA-MB-231 and MDA-MB-453 cells after the knockdown of LINC00673. **c** The colony-forming assays were conducted to examine the proliferation of MDA-MB-231 and MDA-MB-453 cells after the knockdown of LINC00673. **d-e** CCK-8 and colony-forming assays were used to examine the cell proliferation ability in Lv-LINC00673 transfected Hs-578 T cells. **f** Cell cycle analysis of MDA-MB-231 and MDA-MB-453 cells after the knockdown of LINC00673 based on flow cytometry. **g** Flow cytometry analysis of MDA-MB-231 and MDA-MB-453 cells after the knockdown of LINC00673. **h** Expression of bax, bcl-2 and cyclin D1 in MDA-MB-231 and MDA-MB-453 cells after the knockdown of LINC00673, as determined by western blotting. The data are presented as the mean ± the SD of three independent experiments. *P < 0.05, ** *P* < 0.01, and *** *P* < 0.001

To determine the biological functions of LINC00673 in breast cancer cells, siRNAs were designed to silence LINC00673 in MDA-MB-231 and MDA-MB-453 cell lines and lentiviruses were designed to express LINC00673 in the Hs-578 T cell line (Fig. 2a). First, we demonstrated that LINC00673 knockdown suppressed cell viability and colony formation in MDA-MB-231 and MDA-MB-453 cells (Fig. 2b and c), while LINC00673 overexpression had the opposite effects in breast cancer cells (Fig. 2d and e). Next, we found that the suppression of LINC00673 induced cell cycle arrest in the G0/G1 phase, with a reduction in the percentage of cells in S phase compared with that in control cells (Fig. 2f). Compared with the control cells, breast cancer cells with LINC00673 knockdown had significantly increased apoptosis rates (Fig. 2g). Moreover, the knockdown of LINC00673 enhanced bax expression and reduced bcl-2 and cyclin D1 levels in breast cancer cells, confirming that LINC00673 is involved in apoptosis and cell cycle progression (Fig. 2h).

### LINC00673 downregulation suppresses cell proliferation in vivo and is a potential therapeutic target of breast cancer

**Fig. 3:**
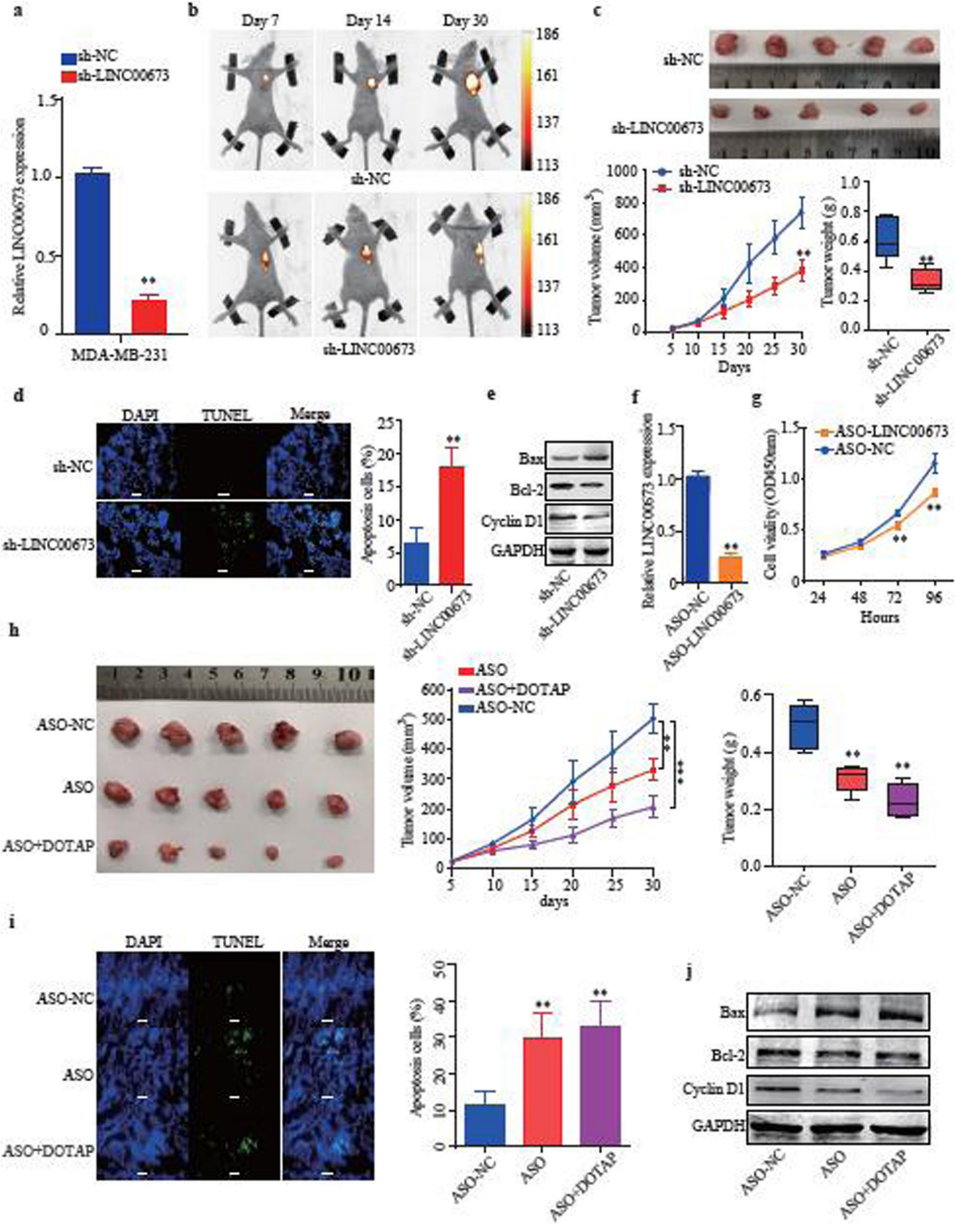
LINC00673 downregulation suppresses cell proliferation in vivo and is a potential therapeutic target of breast cancer. **a** Knockdown of LINC00673 by shRNA in MDA-MB-231 cells was confirmed by qRT-PCR. **b** Luminescence images of subcutaneous tumors in xenograft mouse models bearing tumors generated from MDA-MB-231 cells that were stably transfected with sh-LINC00673 or sh-NC on days 7, 14 and 30 after tumor cell injection. **c** Tumor volume and weight in mice treated with sh-LINC00673 or sh-NC. Tumor volume was calculated every 5 days, *n* = 5. **d** The effect of LINC00673 knockdown on tumor cell apoptosis was determined by TUNEL staining. **e** Protein levels of Bax, Bcl-2 and CyclinD1 in tumor tissues after the knockdown of LINC00673, as analyzed by western blotting assays. **f** qRT-PCR analysis of the relative LINC00673 expression in MDA-MB-231 cells transfected with ASO-LINC00673 (5 μM). The 0 μM group was used as the control. **g** CCK-8 assay detection of cell viability in MDA-MB-231 cells. The concentration of ASO was 5 μM. The 0 μM group was used as the control. **h** Representative ASO treatment tumor images, volumes and weights in the three treatment groups, *n* = 5. **i** The effect of ASO treatment on tumor cell apoptosis was determined by TUNEL staining. **j** Expression of Bax, Bcl-2 and Cyclin D1 in the three treatment groups, as determined by western blotting. The data are presented as the mean ± the SD of three independent experiments. ** *P* < 0.01 and *** *P* < 0.001. Scale bar: 50 μm

To investigate the effects of LINC00673 on breast cancer proliferation in vivo, we inoculated nude mice with MDA-MB-231 cells that stably expressed lentiviral sh-LINC00673 to suppress LINC00673 expression (Fig. 3a). Tumor xenografts with downregulated LINC00673 showed markedly reduced volumes and weights compared to control xenografts (Fig. 3b and c). More apoptosis was observed in cells with downregulated LINC000763 than in control cells (Fig. 3d). Additionally, bax expression was increased while bcl-2 and cyclin D1 expression were reduced in the cells with downregulated LINC000763 compared to that in the control cells (Fig. 3e). Here, we explored the potential role of LINC00673 as a therapeutic target using antisense oligonucleotide (ASO). We designed ASO and confirmed that it decreased LINC00673 expression (Fig. 3f), and the ASO significantly impaired the proliferation of MDA-MB-231 cells (Fig. 3g). To evaluate the efficacy of this anti-LINC00673 targeted therapy, we created a xenograft mouse model. During the treatment process, we observed that the tumor volume and weight were significantly reduced in ASO-treated mice compared with the tumor volume and weight in normal saline-treated control mice. More importantly, 1,2-dioleoyl-3-trimethylammonium-propane (DOTAP) liposomal ASO treatment more effectively inhibited tumor progression than the free ASO treatment (Fig. 3h). An increased number of apoptotic cells (Fig. 3i), increased bax expression and decreased bcl-2 and cyclinD1 expression were observed in the liposomal ASO treatment group compared to the control group (Fig. 3j). Previous research suggested that ASO may cause toxicity in the liver or in other internal organs [37, 38]. TUNEL and H&E staining revealed mild cell apoptosis and necrosis in the organs, and the biochemical parameters were similar between the treatment and control groups, which suggests that the ASO toxicity was limited (Additional file 2: Figure S2). Together, these results suggested that LINC00673 significantly promoted tumor growth and that the liposomal delivery of an ASO targeted to LINC00673 has great potential as a novel antitumor therapeutic agent.

### LINC00673 promotes breast cancer cell proliferation by modulating the MARK4 and Hippo signaling pathways

**Fig. 4:**
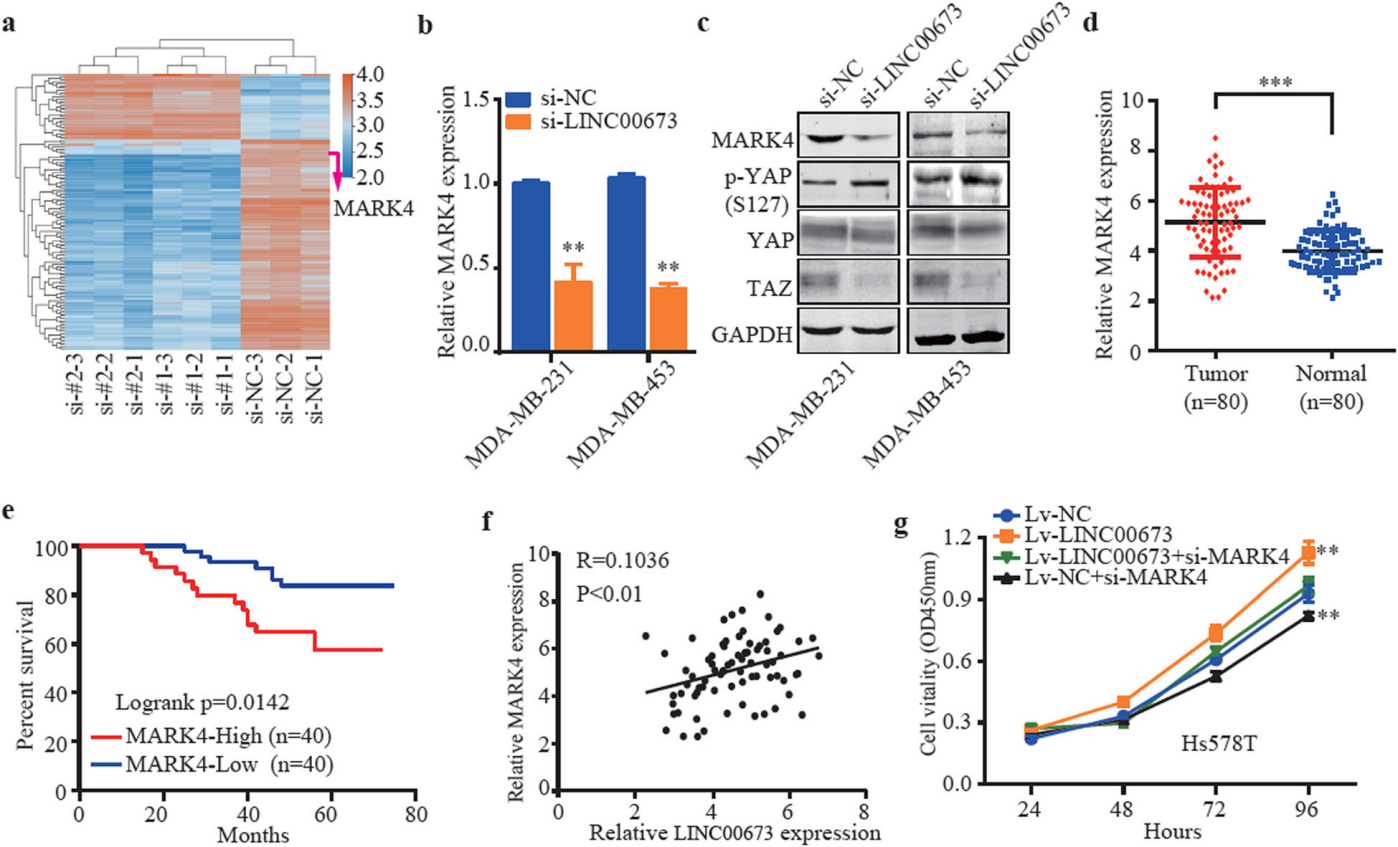
LINC00673 promotes breast cancer cell proliferation by modulating the MARK4 and Hippo signaling pathways. **a** Expression heatmap of significantly differentially expressed transcripts after the RNA transcriptome sequencing analysis following LINC00673 knockdown. The red color indicates genes that were upregulated compared with the control cells, and the blue color indicates genes that were downregulated compared with the control cells. **b** The expression of MARK4 after the knockdown of LINC00673 was measured by using qRT-PCR. **c** Western blotting analysis of MARK4, p-YAP, YAP and TAZ in the indicated cells. **d** The expression of MARK4 was determined by qRT-PCR in 80 paired breast cancer tissues and adjacent normal tissues. **e** Kaplan-Meier analyses of the correlations between MARK4 expression and OS in 80 breast cancer patients. **f** A positive correlation was confirmed between LINC00673 and MARK4 by Pearson correlation analysis. **g** Growth ability of Hs-578 T cells after cotransfection with Lv-LINC00673, si-MARK4, as determined by CCK-8 assays. The data are presented as the mean ± the SD of three independent experiments. ** *P* < 0.01 and *** *P* < 0.001

RNA sequencing was performed to investigate the potential molecular mechanism of LINC00673 in breast cancer progression. MARK4, a member of the microtubule affinity regulating kinase (MARK) family, has been reported to promote proliferation via the Hippo signaling pathway in breast cancer cells [39]. Sequencing revealed that MARK4 mRNA expression was significantly reduced in the MDA-MB-453 cells in which LINC00673 was downregulated by siRNA compared to that in control cells (Fig. 4a). We found that compared to control cells, breast cancer cells with LINC00673 knockdown had significantly reduced MARK4, YAP, and TAZ expression and increased YAP phosphorylation levels in the Hippo signaling pathway (Fig. 4b and c). Moreover, we found that MARK4 was significantly overexpressed in human breast cancer tissues with shorter OS (Fig. 4d and e), and a positive correlation was found between LINC00673 and MARK4 (Fig. 4f). The results of CCK-8 assays showed that cotransfection could partially rescue the Lv-LINC00673 elevated growth ability in Hs-578 T cells (Fig. 4g). In summary, we demonstrated that LINC00673 promotes breast cancer cell proliferation via the Hippo signaling pathway by regulating MARK4.

### LINC00673 regulates MARK4 expression by competing for miR-515-5p

**Fig. 5:**
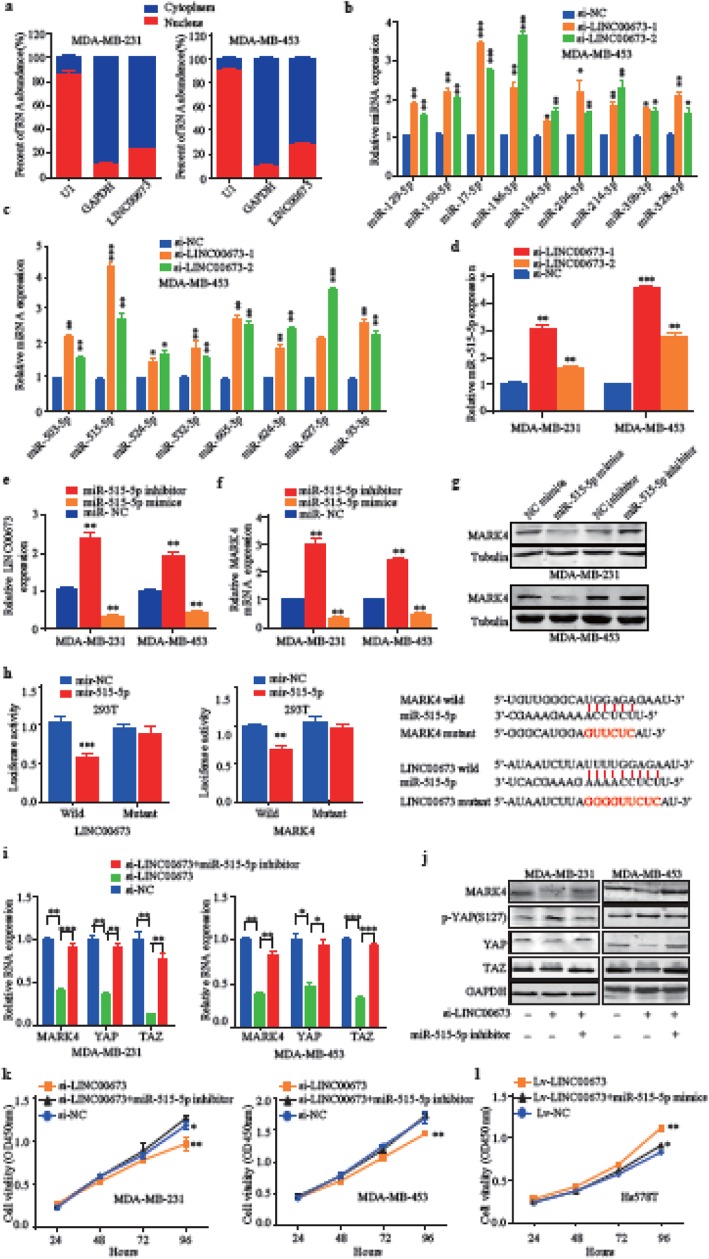
LINC00673 regulates MARK4 expression by competing for miR-515-5p. **a** The subcellular distribution of LINC00673 in MDA-MB-231 and MDA-MB-453 cells. GAPDH was used as the cytoplasmic control, and U1 served as the nuclear control. **b-c** The relative expression of miRNAs was determined by qRT-PCR after the knockdown of LINC00673. **d** The expression of miR-515-5p was measured after the knockdown of LINC00673 by using qRT-PCR in MDA-MB-231 and MDA-MB-453 cells. **e** Expression of LINC00673 in miR-515-5p mimics or inhibitor transfected MDA-MB-231 and MDA-MB-453 cells, as determined by qRT-PCR. **f-g** Elevation and depression of miR-515-5p was inversely related to MARK4 expression, as determined by qRT-PCR and western blotting. **h** Luciferase reporter assays were used to verify the targeted binding between LINC00673 or MARK4 3’untranslated region (UTR) and miR-515-5p. **i** The expression of MARK4, YAP and TAZ in MDA-MB-231 and MDA-MB-453 cells transfected with LINC00673 siRNA or cotransfected with a LINC00673 siRNA and an miR-515-5p inhibitor, as determined by qRT-PCR. **j** Western blot analysis of MARK4, p-YAP, YAP and TAZ expression levels in MDA-MB-231 and MDA-MB-453 cells transfected with LINC00673 siRNA or cotransfected with LINC00673 siRNA and miR-515-5p inhibitor. **k-l** CCK-8 assays were used to examine the cell proliferation ability after LINC00673 knockdown in MDA-MB-231 and MDA-MB-453 cells transfected with miR-515-5p inhibitors and LINC00673-overexpressing cells transfected with miR-515-5p mimics. The data are presented as the mean ± the SD of three independent experiments. *P < 0.05, ** *P* < 0.01, and *** *P* < 0.001

Recent findings indicate that lncRNAs can exert their function in different compartments of the cell. Most of the lncRNAs that have been investigated thus far act in the nucleus by regulating gene expression [40]. However, lncRNAs also act as ceRNAs to regulate mRNA degradation and translation in the cytoplasm [41]. Thus, we first assessed the cellular localization of LINC00673 by measuring its expression in the nucleus and cytoplasm in MDA-MB-453 and MDA-MB-231 cells. As shown in Fig. 5a, LINC00673 was mainly found in the cytoplasm of breast cancer cells. Therefore, we examined whether LINC00673 might be involved in cross-talk with any miRNAs in breast cancer. We used both the LncBook (https://bigd.big.ac.cn/lncbook) and TargetScan (http://www.targetscan.org/) databases to search for miRNAs that could be closely associated with LINC00673 and MARK4, and together these databases identified 258 miRNAs that may act as targets of LINC00673 and MARK4. Most of these miRNAs have unknown functions, therefore, we selected 40 miRNAs that have been reported to be involved in numerous cancers for further study (Additional file 4: Table S2). Among these miRNAs, we verified that the expression of 18 miRNAs were significantly increased by the knockdown of LINC00673 (Fig. 5b and c). Simultaneously, we noticed that one of these candidate miRNAs, miR-515-5p, has been found to dramatically inhibit breast cancer cell proliferation and to control cancer cell migration through MARK4 regulation in breast cancer [42, 43]. Therefore, we examined whether LINC00673 could interact with miR-515-5p to regulate MARK4. We verified that the knockdown of LINC00673 negatively regulated miR-515-5p in both MDA-MB-231 and MDA-MB-453 cells (Fig. 5d). The upregulation and downregulation of miR-515-5p inversely affected LINC00673 expression (Fig. 5e). Then, we showed that miR-515-5p negatively regulated MARK4 mRNA and protein expression (Fig. 5f and g). We next explored whether miR-515-5p could directly bind to LINC00673 and MARK4. Dual-luciferase assays indicated that there was a significant reduction in luciferase activities after the cotransfection of miR-515-5p mimics and a wild-type LINC00673 or MARK4 reporter vector, but this reduction was not observed with the transfection of mutant LINC00673 and MARK4 reporter vectors (Fig. 5h). Finally, the treatment of MDA-MB-231 and MDA-MB-453 cells with si-LINC00673 and miR-515-5p inhibitors attenuated the reduction in MARK4, YAP and TAZ mRNA transcript levels caused by LINC00673 knockdown (Fig. 5i). Notably, the reduction in MARK4 and YAP/TAZ and the increase phosphorylated YAP protein expression was also abolished by miR-515-5p inhibitors in breast cancer cells transfected with si-LINC00673 (Fig. 5j). We next determined whether LINC00673 induced breast cancer cell proliferation through the miR-515-5p. The results of CCK-8 assays showed that the knockdown of LINC00673 expression dramatically reduced the proliferative capacity of breast cancer cells. As expected, in the cells cotransfected with si-LINC00673 and the miR-515-5p inhibitor, these effects were abolished (Fig. 5k). Similarly, the cotransfection of cells with Lv-LINC00673 and the miR-515-5p mimic attenuated the LINC00673-mediated increasein cell proliferation (Fig. 5l). In summary, these results showed that LINC00673 promoted tumor cell growth at least in part by acting as a ceRNA for miR-515-5p and thus regulating the MARK4/Hippo signaling pathways.

### YY1 activates LINC00673 expression in breast cancer cells

**Fig. 6:**
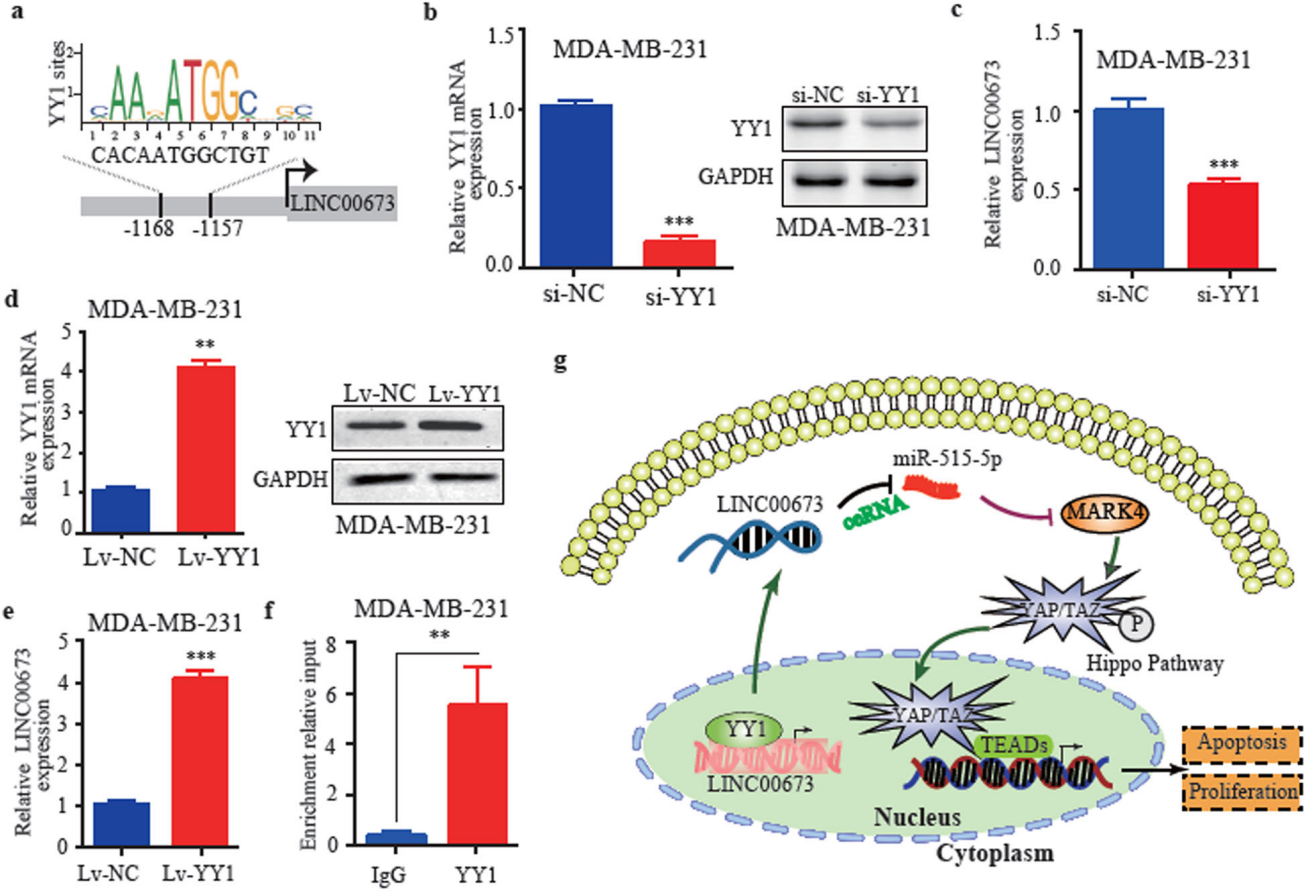
YY1 activates LINC00673 expression in breast cancer cells. **a** YY1 binding motif and the prediction of YY1 binding sites within the promoter region of LINC00673 provided by the JASPAR database. **b** The knockdown efficiency of YY1 in MDA-MB-231 cells was determined by qRT-PCR and western blotting. **c** qRT-PCR analysis of LINC00673 expression in MDA-MB-231 cells after transfection with YY1 siRNA and the negative control. **d** The overexpression efficiency of YY1 in MDA-MB-231 cells was determined by qRT-PCR and western blot analysis. **e** qRT-PCR analysis of LINC00673 expression in MDA-MB-231 cells after transfection with Lv-YY1 and the negative control. **f** qRT-PCR of the ChIP products validating the binding capacity of YY1 to the LINC00673 promoter. **g** The mechanism of the regulatory network and function of LINC00673. LINC00673 promoted proliferation, induced apoptosis in breast cancer cells which could be enhanced by YY1 and acted as a ceRNA for miR-515-5p to regulate MARK4 and inactivate the Hippo signaling pathway. The data are presented as the mean ± the SD of three independent experiments. ** *P* < 0.01 and *** *P* < 0.001

To further examine the transcriptional regulation model of LINC00673 in breast cancer, we searched the TRANSFAC (http://gene-regulation.com/) and JASPAR (http://jaspar.genereg.net/) databases to identify transcription factors that may regulate LINC00673. The transcription factor Yin Yang 1 (YY1) was predicted by both the TRANSFAC and JASPAR data bases with high scores (Additional file 5: Table S3). The predicted binding sites of YY1 in the LINC00673 promoter sequence are illustrated in Fig. 6a. To explore whether LINC00673 is a downstream target of YY1, we knocked down YY1 by siRNA in MDA-MB-231 cells, which led to a significant decrease in LINC00673 expression (Fig. 6b and c). Furthermore, the overexpression of YY1 significantly elevated LINC00673 expression in MDA-MB-231 cells (Fig. 6d and e). Moreover, ChIP assays showed that the LINC00673 promoter was specifically pulled down by a YY1-specific antibody but not the control antibody (Fig. 6f). Taken together, these findings suggest that YY1 is a bona fide transcriptional activator of LINC00673.

## Discussion

With the development of techniques for sequencing entire genomes, increasing number of molecular mechanisms in breast cancer have been described [44, 45]. LncRNAs have been shown to play important roles and to act through multiple mechanisms, including as ceRNAs, in a variety of cancers [46–48]. There is still some controversy regarding the role of LINC00673 in carcinogenesis, numerous studies have described LINC00673 as either a tumor suppressor or promoter [11–15]. Although previous studies have also identified the oncogenic role of LINC00673 was upregulation in breast cancer tissues and related with patients’ prognosis [19], very little is known about the molecular mechanisms of LINC00673 in breast cancer carcinogenesis. To this end, in the current study, we confirmed the oncogenic function of LINC00673, as it enhanced the proliferative capacity of breast cancer cells in a series of functional experiments both in vitro and in vivo. In addition, we demonstrated that LINC00673 expression was positively correlated with tumor size and Ki67 status. Of note, Xia E et al. reported that LINC00673 could influence B7-H6 expression to enhance cell migration, invasion and epithelial-mesenchymal transition (EMT) in breast cancer [19]. However, no significant association was found between LINC00673 and lymph node metastasis (LNM), tumor-node-metastasis (TNM) stages in our study. Due to the randomness of clinical samples that may lead to different experimental results, the number of patients included in the validated cohort should be increased to provide more convincing results. Given the significant effects of LINC00673 on breast cancer progression, the mechanism related to LINC00673 urgently needs to be explored.

Here, we used RNA-seq to identify the regulatory networks of mRNAs and noncoding RNAs in breast cancer. Among the results from the analysis of potential mRNA interactions, MARK4 sparked our interest since it is associated with tumorigenesis [49, 50]. Previous studies showed that MARK4 attenuates the proliferation and migration of MDA-MB-231 cells via the Hippo signaling pathway [39]. The Hippo pathway plays a critical role in regulating multiple aspects of growth at both the cellular and organ levels [20]. The main factors in this pathway are YAP and TAZ, which associate with the TEAD family of transcription factors [51, 52]. When the Hippo pathway turned on that limits tissue growth and cell proliferation by phosphorylating and inhibiting YAP/TAZ. On the contrary, once the Hippo pathway is deactivated, YAP/TAZ are dephosphorylated and translocate into the nucleus, where they bind to TEAD and enhance proliferation, restrain apoptosis, and promote the migration of cancer cells [20]. In our study, we observed that LINC00673 induced the expression of MARK4, which was accompanied by alterations in YAP phosphorylation and total YAP protein levels, showing that LINC00673 inhibits the Hippo signaling pathway. Moreover, we found that LINC00673 was predominantly distributed in the cytoplasm of the cell, indicating that it plays a role in posttranscriptional regulation. There is increasing evidence that lncRNAs can function as ceRNAs for miRNAs in cancer [53]. Thus, our investigations, such as luciferase activity assays and rescue experiments, further confirmed that LINC00673 functions as a ceRNA by binding miR-515-5p to regulate MARK4 and then inhibits the Hippo pathway. All of these results led us to propose the existence of a regulatory network in which miRNAs and lncRNAs interact with each other to coregulate the expression and function of MARK4. The transcription factor YY1 is associated with cell differentiation, apoptosis and tumorigenesis [54]. The abnormal expression of YY1 contributes to several human cancers and correlates with poor prognosis [55, 56]. However, the function of YY1 in breast cancer remains controversial [57–59]. In the current study, we found that YY1 may act as an oncogene that can directly bind to the LINC00673 promoter region. Despite these findings, we need to conduct more experiments in vivo or in vitro to deeply investigate the interactions between YY1 and LINC00673.

LncRNAs act either as tumor promoters or suppressors, suggesting that the manipulation of their regulation may have potential for breast cancer treatment. Various methods have been developed to interfere with the functions of lncRNAs, such as using ASOs, the liposomal or nanoparticle-mediated delivery of various treatments, and emerging gene editing technology with CRISPR Cas9 systems and ncRNA editing in cancers [60, 61]. Compared with traditional RNA interference technologies, such as siRNA, ASOs have some other advantages for clinical practice, which include a longer half-life, highly efficient cellular uptake and stronger silencing effects [62–64]. Xing et al. showed that the therapeutic delivery of antisense locked nucleic acids (LNAs) specific to the lncRNA BCAR4 effectively suppressed metastasis in a breast cancer mouse model [65]. Further, the suppression of the lncRNA Malat1 using an ASO resulted in up to an 80% inhibition of metastasis in a luminal B breast cancer mouse model [66]. Liposomes have been widely investigated as drug carriers for improving the delivery of therapeutic agents to specific sites in the body and were almost immediately explored for cancer treatment. Lipophilic and hydrophilic drugs can be incorporated into the lipid membrane or inner aqueous space of liposomes respectively. While modified uncharged nucleotides can be delivered by neutral or slightly charged liposomes, native negatively charged ASO, siRNA, or DNA molecules required cationic liposomes [67]. In the present study, to investigate the therapeutic potential of LINC00673 in breast cancer, 1,2-dioleoyl-3-trimethylammonium-propane (DOTAP) cationic liposomes were used to deliver ASO-LINC00673. As expected, ASO successfully inhibited the progression of breast cancer in vivo*.* Notably, intravenous treatment with liposomal ASO was much more efficient in limiting tumor growth than treatment with free ASO. Thus, the future development of lncRNAs as potential therapeutics in the breast cancer, as well as in other cancers, seems promising.

## Conclusions

In all, we showed that LINC00673 is activated by YY1 and acts as a sponge for miR-515-5p, regulating MARK4, inactivating the Hippo signaling pathway, and resulting in tumor progression (Fig. 6g). More importantly, LINC00673 is a potential therapeutic target for treating breast cancer.

## Supplementary information

**Additional file 1: Figure S1.** LINC00673 is highly expressed in breast cancer tissues. (a) LINC00673 data downloaded from the MiTranscriptome database. (b) Expression of Linc00673 in 950 breast cancer tissues and 107 normal breast tissues (TCGA). *** *P* < 0.001.

**Additional file 2: Figure S2.** Potential therapeutic role of LINC00673 in breast cancer progression. (a) Effect of ASO on apoptosis in mouse organs. (b) H&E staining and sections were observed under an Olympus microscope. (c) Serum chemistry markers of liver and renal function in the 0.9% normal saline and ASO treatment groups. GPT: glutamic pyruvic transaminase; ALP: alkaline phosphatase; GGT: gamma-glutamyl transpeptidase; BUN: blood urea nitrogen; CRE: serum creatinine; and TBIL: total bilirubin.*P < 0.05, scale bar: 50 μm.

**Additional file 3: Table S1.** Sequences of the primer pairs for q-PCR and sequences of RNAi for transfection.

**Additional file 4: Table S2.** miRNAs associated with LINC00673 and MARK4, as predicted by LncBook and TargetScan.

**Additional file 5: Table S3.** Transcription binding site prediction was conducted by TRANSFAC and JASPAR.

## Data Availability

The authors declare that the data supporting the findings of this study are available within the article and its supplementary information files.

## Abbreviations

ASO: Antisense oligonucleotide
ceRNA: Competing endogenous RNA
ChIP: Chromatin immunoprecipitation
DOTAP: 1,2-dioleoyl-3-trimethylammonium-propane
LINC00673: Long intergenic non-protein coding RNA 673
LncRNA: Long non-coding RNA
MARK4: Microtubule affinity regulating kinase 4
TAZ: Transcriptional coactivator with PDZ-binding motif
YAP: Yes-associated protein 1
YY1: Yin Yang 1
