# Correction to: LINC00673 is activated by YY1 and promotes the proliferation of breast cancer cells via the miR-515-5p/MARK4/Hippo signaling pathway

**DOI:** 10.1186/s13046-020-01645-4

**Published:** 2020-08-11

**Authors:** Kun Qiao, Shipeng Ning, Lin Wan, Hao Wu, Qin Wang, Xingda Zhang, Shouping Xu, Da Pang

**Affiliations:** 1 Department of Breast Surgery, Harbin Medical University Cancer Hospital, 150 Haping Road, Harbin, 150086 China; 2 Heilongjiang Academy of Medical Sciences, 157 Baojian Road, Harbin, 150086 China

**Correction to: J Exp Clin Cancer Res 38, 418 (2019)**

**https://doi.org/10.1186/s13046-019-1421-7**

Following publication of the original article [1], the authors identified several errors as below:

1. In Table 1, some data needs to be revised:

Under ‘Age < 50 & LINC00673 expression’: Low 24, High 14, *P* value = 0.446 should be corrected to Low 17, High 21, *P* value = 0.370.

Under ‘Tumor size ≤2cm & LINC00673 expression’: Low 20, High 11, *P* value = 0.024 should be corrected to Low 21, High 10, *P* value = 0.011.

Under ‘Negative PR expression’: 45 should be corrected to 48.

As a result, a sentence in the section ‘LINC00673 is upregulated in breast cancer tissues and cell lines’ should be changed as well.

The sentence currently reads:

LINC00673 expression was positively correlated with tumor size (*P* = 0.024), and Ki67 status (*P* = 0.019).

The sentence should read:

LINC00673 expression was positively correlated with tumor size (*P* = 0.011), and Ki67 status (*P* = 0.019).

2. In the ‘Methods, Statistical analysis’ section, the term ‘Spearman’s’ had been incorrectly used.

The sentence currently reads:

The correlation between LINC00673 and MARK4 expression was analyzed using Spearman’s correlation test.

The sentence should read:

The correlation between LINC00673 and MARK4 expression was analyzed using Pearson’s correlation test.

3. An error was identified in the figure legend of Fig. 4a.

The sentence currently reads:

The red color indicates genes that were upregulated compared with the control cells, and the blue color indicates genes that were downregulated compared with the control cells.

The sentence should read:

The red to blue color gradient indicates high to low expression levels, respectively.

4. In the section ‘LINC00673 regulates MARK4 expression by competing for miR-515-5p’, the number ‘18’ needs to be corrected to ‘17’ in the sentence ‘Among these miRNAs, we verified that the expression of 18 miRNAs were significantly increased by the knockdown of LINC00673 (Fig. 5b and c)’.

The correct sentence should read:

Among these miRNAs, we verified that the expression of 17 miRNAs were significantly increased by the knockdown of LINC00673 (Fig. 5b and c).

5. An error was identified in the Fig. 6e, which should have indicated LINC00673 expression in MDA-MB-231 cells after transfection with Lv-YY1, was mistakenly displayed as a duplicate of Fig. 6d, due to the authors’ carelessness during the layout of figure.
6. An error was identified in Fig. 6g. The phosphorylated YAP/TAZ in cytoplasm should have been non-phosphorylated YAP/TAZ. In addition, to better understand the mechanism of the regulatory network and function of LINC00673, the authors added up and down arrows in Fig. 6g. In the figure legend of Fig. 6g, the second sentence contains an error. The authors had incorrectly used the term ‘induced’, and it should be changed to ‘reduced’. As a result, the second sentence in the figure legend of Fig. 6g ‘LINC00673 promoted proliferation, induced apoptosis in breast cancer cells which could be enhanced by YY1 and acted as a ceRNA for miR-515-5p to regulate MARK4 and inactivate the Hippo signaling pathway.’ should be changed to ‘LINC00673 promoted proliferation, reduced apoptosis in breast cancer cells which could be enhanced by YY1 and acted as a ceRNA for miR-515-5p to regulate MARK4 and inactivate the Hippo signaling pathway.’
7. Figure 5 is not clear in the article, and should be replaced with an updated one with high resolution.
8. Additional File 1 Figure S1 and Additional File 2 Figure S2 were mistakenly displayed as Fig. 1 and Fig. 2.

The updated figures and tables are shown below:

**Table 1:** Correlation between LINC00673 expression and the clinicopathological features of breast cancer

Characteristics	No. (*n* = 80)	LINC00673 expression		*P*-value
		Low (*n* = 40)	High (*n* = 40)	
Age				0.370
< 50	38	17	21	
≥ 50	42	23	19	
Tumor size				0.011^*^
≤ 2 cm	31	21	10	
> 2 cm	49	19	30	
LNM				0.097
Negative	27	17	10	
Positive	53	23	30	
TNM stage				0.089
I-II	74	39	35	
III-IV	6	1	5	
ER expression				0.485
Negative	29	16	13	
Positive	51	24	27	
PR expression				
Negative	48	23	25	0.648
Positive	32	17	15	
Her-2 expression				0.368
Negative	44	24	20	
Positive	36	16	20	
Ki67 expression				0.019^*^
≤ 14%	28	19	9	
> 14%	52	21	31	

*LNM* lymph node metastasis, *P < 0.05

**Fig. 5:**
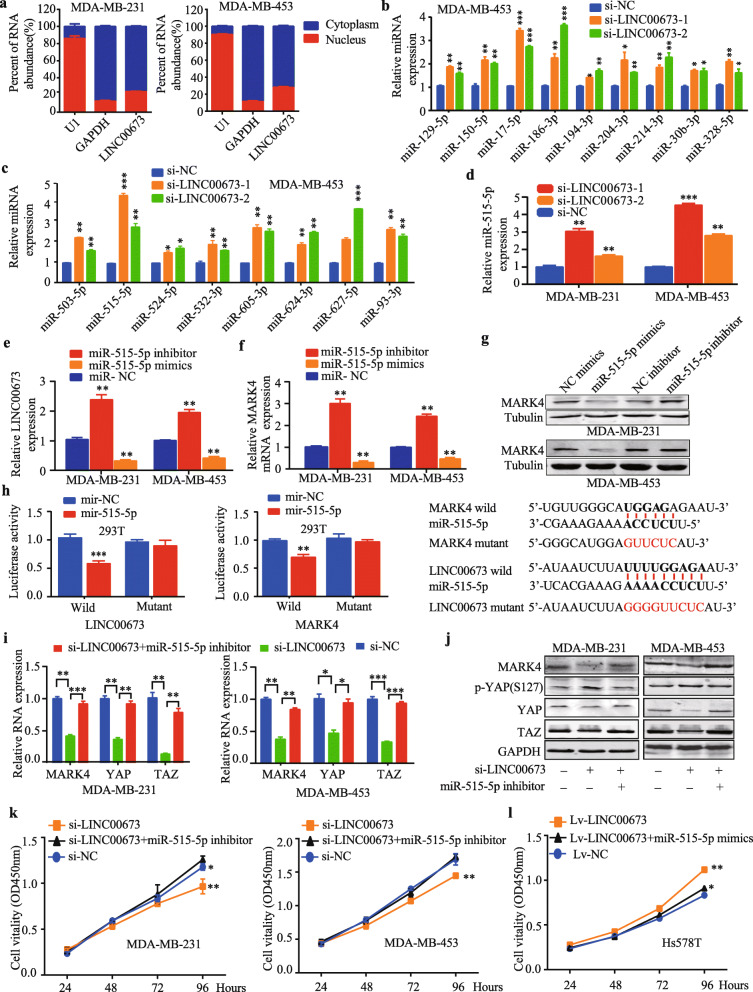
LINC00673 regulates MARK4 expression by competing for miR-515-5p. **a** The subcellular distribution of LINC00673 in MDA-MB-231 and MDA-MB-453 cells. GAPDH was used as the cytoplasmic control, and U1 served as the nuclear control. **b**-**c** The relative expression of miRNAs was determined by qRT-PCR after the knockdown of LINC00673. **d** The expression of miR-515-5p was measured after the knockdown of LINC00673 by using qRT-PCR in MDA-MB-231 and MDA-MB-453 cells. **e** Expression of LINC00673 in miR-515-5p mimics or inhibitor transfected MDA-MB-231 and MDA-MB-453 cells, as determined by qRT-PCR. **f**-**g** Elevation and depression of miR-515-5p was inversely related to MARK4 expression, as determined by qRT-PCR and western blotting. **h** Luciferase reporter assays were used to verify the targeted binding between LINC00673 or MARK4 3’untranslated region (UTR) and miR-515-5p. **i** The expression of MARK4, YAP and TAZ in MDA-MB-231 and MDA-MB-453 cells transfected with LINC00673 siRNA or cotransfected with a LINC00673 siRNA and an miR-515-5p inhibitor, as determined by qRT-PCR. **j** Western blot analysis of MARK4, p-YAP, YAP and TAZ expression levels in MDA-MB-231 and MDA-MB-453 cells transfected with LINC00673 siRNA or cotransfected with LINC00673 siRNA and miR-515-5p inhibitor. **k**-**l** CCK-8 assays were used to examine the cell proliferation ability after LINC00673 knockdown in MDA-MB-231 and MDA-MB-453 cells transfected with miR-515-5p inhibitors and LINC00673-overexpressing cells transfected with miR-515-5p mimics. The data are presented as the mean ± the SD of three independent experiments. *P < 0.05, ** *P* < 0.01, and *** *P* < 0.001

**Fig. 6:**
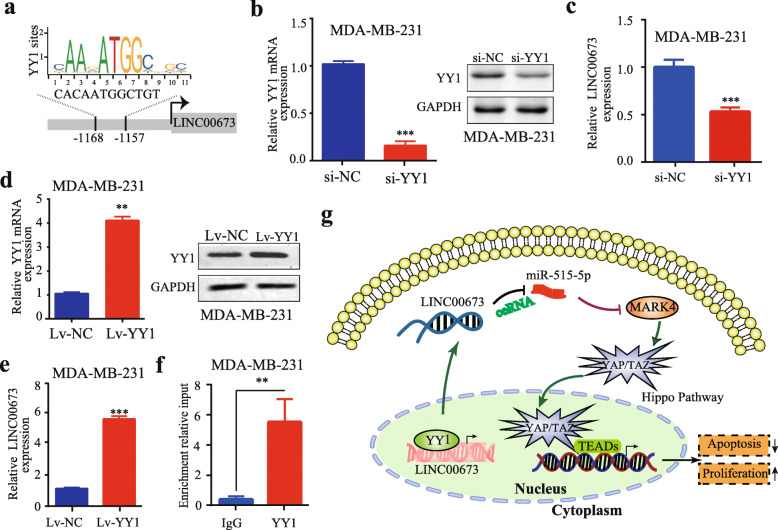
YY1 activates LINC00673 expression in breast cancer cells. **a** YY1 binding motif and the prediction of YY1 binding sites within the promoter region of LINC00673 provided by the JASPAR database. **b** The knockdown efficiency of YY1 in MDA-MB-231 cells was determined by qRT-PCR and western blotting. **c** qRT-PCR analysis of LINC00673 expression in MDA-MB-231 cells after transfection with YY1 siRNA and the negative control. **d** The overexpression efficiency of YY1 in MDA-MB-231 cells was determined by qRT-PCR and western blot analysis. **e** qRT-PCR analysis of LINC00673 expression in MDA-MB-231 cells after transfection with Lv-YY1 and the negative control. **f** qRT-PCR of the ChIP products validating the binding capacity of YY1 to the LINC00673 promoter. **g** The mechanism of the regulatory network and function of LINC00673. LINC00673 promoted proliferation, reduced apoptosis in breast cancer cells which could be enhanced by YY1 and acted as a ceRNA for miR-515-5p to regulate MARK and inactivate the Hippo signaling pathway. The data are presented as the mean ± the SD of three independent experiments. ** *P* < 0.01 and *** *P* < 0.001

## Supplementary information

**Additional file 1 **: **Figure S1.** LINC00673 is highly expressed in breast cancer tissues. (a) LINC00673 data downloaded from the MiTranscriptome database. (b) Expression of LINC00673 in 950 breast cancer tissues and 107 normal breast tissues (TCGA). *** *P* < 0.001.

**Additional file 2 **: **Figure S2.** Potential therapeutic role of LINC00673 in breast cancer progression. (a) Effect of ASO on apoptosis in mouse organs. (b) H&E staining and sections were observed under an Olympus microscope. (c) Serum chemistry markers of liver and renal function in the 0.9% normal saline and ASO treatment groups. GPT: glutamic pyruvic transaminase; ALP: alkaline phosphatase; GGT: gamma-glutamyl transpeptidase; BUN: blood urea nitrogen; CRE: serum creatinine; and TBIL: total bilirubin.*P < 0.05, scale bar: 50 μm.
