# Supplementary figures and images for: Correction to: LINC00673 is activated by YY1 and promotes the proliferation of breast cancer cells via the miR-515-5p/MARK4/Hippo signaling pathway
Source: J Exp Clin Cancer Res. 2020 Aug 11;39:154. doi: 10.1186/s13046-020-01645-4 (PMC7418315; doi:10.1186/s13046-020-01645-4)

**a**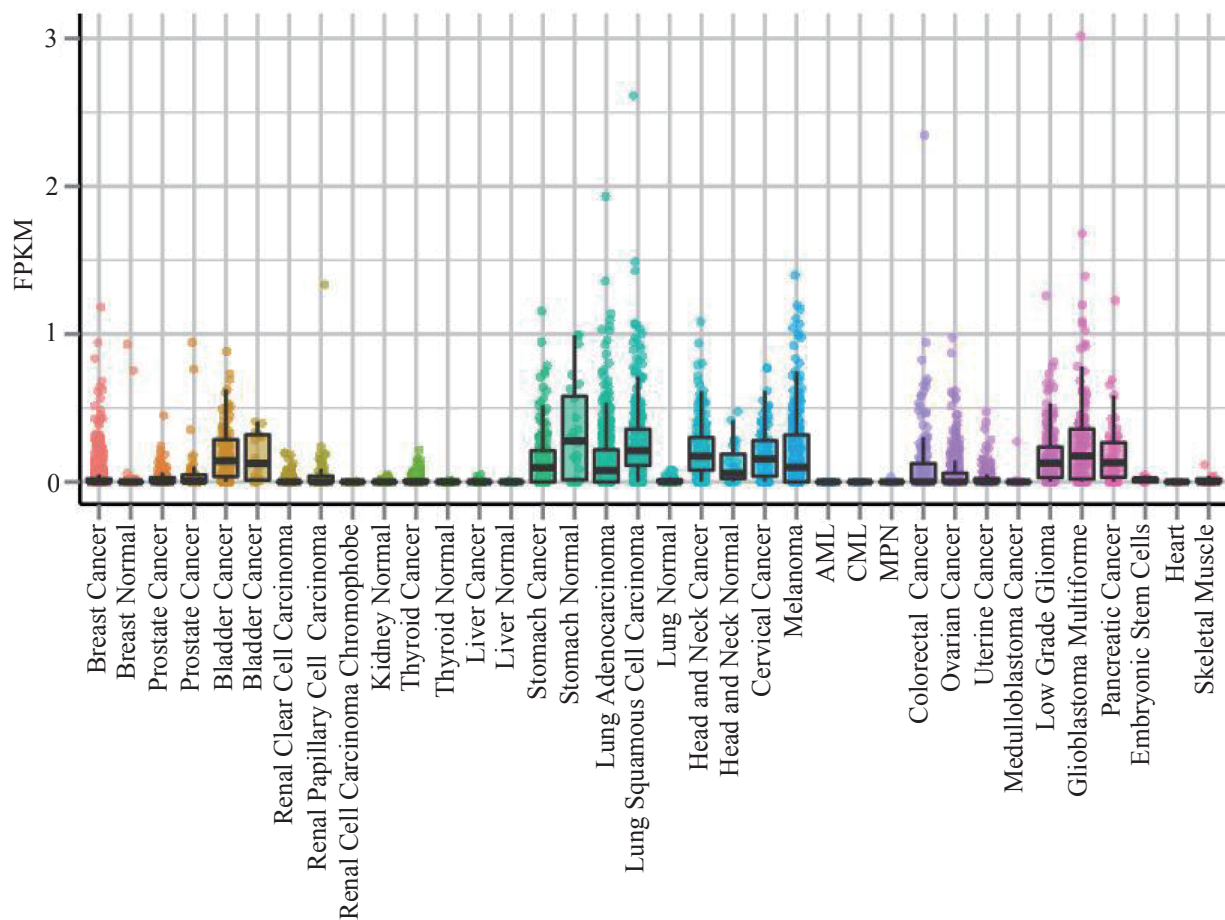**b**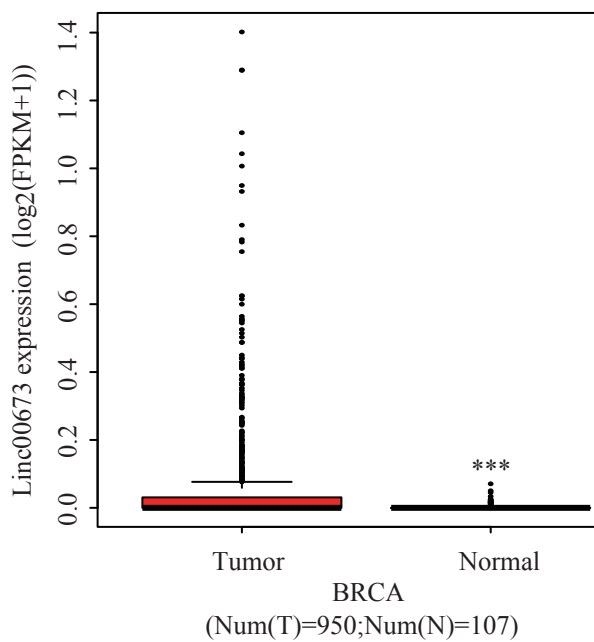

Supplement: Supplementary file 1 — Additional file 1 : Figure S1. LINC00673 is highly expressed in breast cancer tissues. (a) LINC00673 data downloaded from the MiTranscriptome database. (b) Expression of LINC00673 in 950 breast cancer tissues and 107 normal breast tissues (TCGA). *** P < 0.001. [file 13046_2020_1645_MOESM1_ESM.pdf]

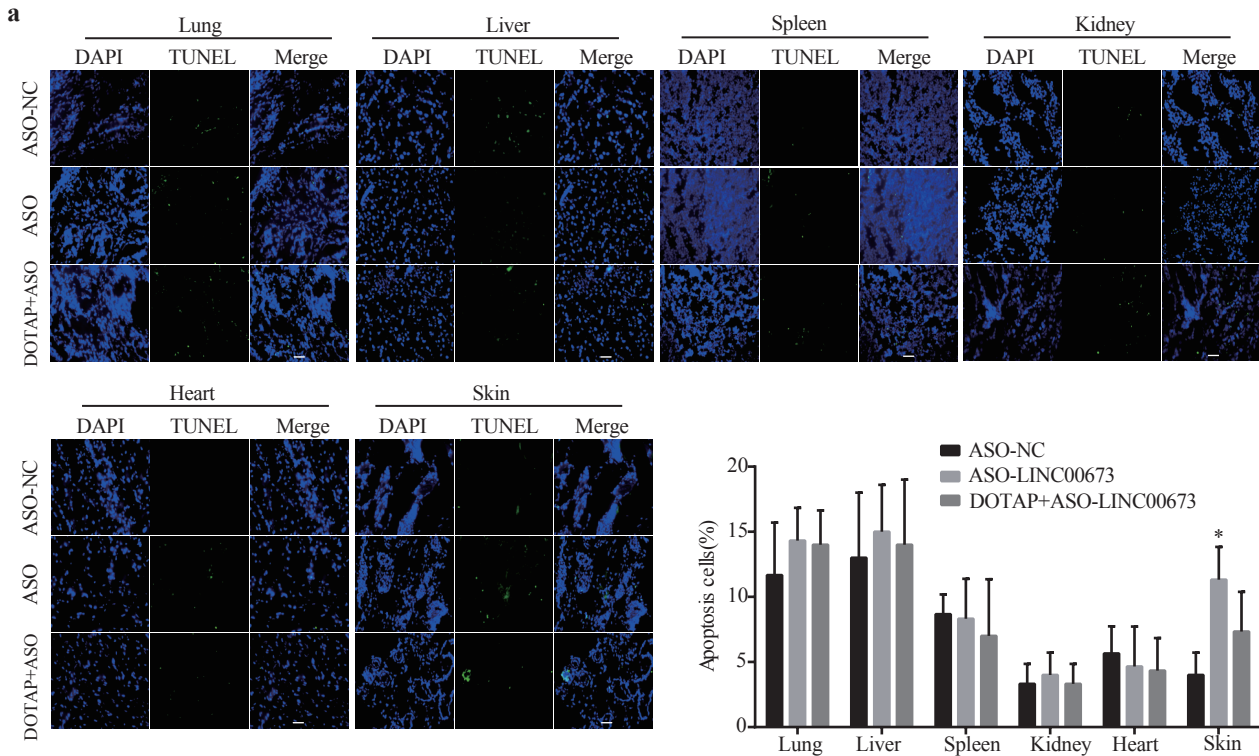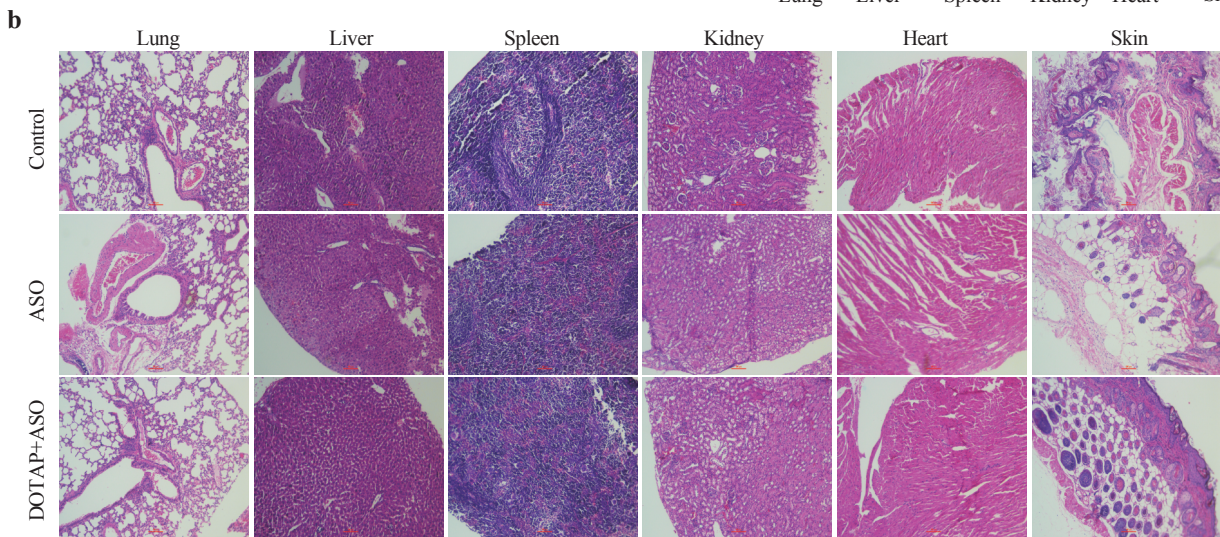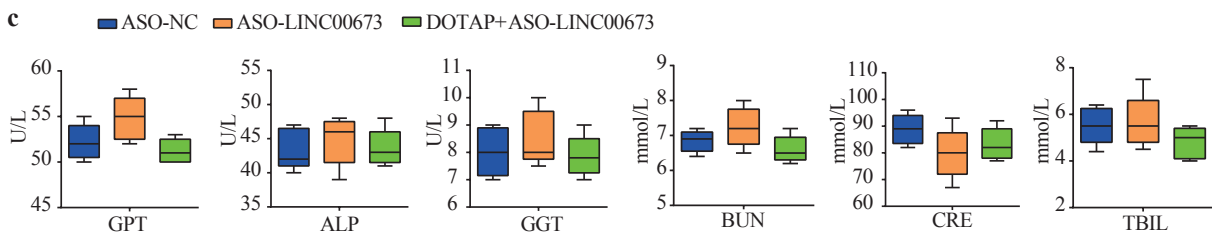

Supplement: Supplementary file 2 — Additional file 2 : Figure S2. Potential therapeutic role of LINC00673 in breast cancer progression. (a) Effect of ASO on apoptosis in mouse organs. (b) H&E staining and sections were observed under an Olympus microscope. (c) Serum chemistry markers of liver and renal function in the 0.9% normal saline and ASO treatment groups. GPT: glutamic pyruvic transaminase; ALP: alkaline phosphatase; GGT: gamma-glutamyl transpeptidase; BUN: blood urea nitrogen; CRE: serum creatinine; and TBIL: total bilirubin.*P < 0.05, scale bar: 50 μm. [file 13046_2020_1645_MOESM2_ESM.pdf]
